# Efficacy of acoustic stimulation techniques on cognitive functions in individuals with Alzheimer’s disease—a scoping review

**DOI:** 10.1186/s13195-024-01544-2

**Published:** 2024-08-01

**Authors:** Leelavathi Thamizhmani, Kanaka Ganapathy, Hari Prakash Palaniswamy, Divya Sussana Patil, Suzanne Carolyn Purdy

**Affiliations:** 1 Department of Speech and Hearing, Manipal College of Health Professions, Manipal Academy of Higher Education, Manipal, India; 2 Centre for Evidence-Informed Decision-Making, Department of Health Information, Prasanna School of Public Health, Manipal Academy of Higher Education, Manipal, India; 3 School of Psychology (Speech Science), Faculty of Science, The University of Auckland, Auckland, New Zealand

**Keywords:** Auditory stimulation, Gamma entrainment, Alzheimer’s disease, Mild cognitive impairment

## Abstract

**Background:**

Alzheimer's disease (AD) is a progressive neurodegenerative disorder that severely affects cognitive functions and social behaviors, leading to a significant decline in an individual’s quality of life. Auditory processing deficits often precede the clinical symptoms of AD, prompting interest in auditory-based interventions as potential treatments. This scoping review aimed to compile the existing evidence on active and passive auditory-based interventions for individuals with AD and its prodromal stages.

**Method and results:**

This scoping review followed Arksey and O’Malley's five-step framework to identify the existing evidence on auditory-based interventions for AD. Four databases (PubMed, Web of Science, CINAHL, and Embase) were used to search for studies on auditory stimulation techniques to treat cognitive decline in AD patients. In total, 14 studies were included in the analysis. Seven studies explored active auditory stimulation techniques, such as the Brain Fitness Program (BrainHQ), aiming to improve cognitive function in individuals with Mild Cognitive Impairment (MCI). The other seven studies focused on passive auditory stimulation, often combined with other sensory stimuli such as light or tactile inputs. Passive stimulation studies have focused mainly on Gamma Entrainment Using Sensory Stimulation (GENUS). The intervention frequency and duration varied across studies, ranging from one session lasting 8 h to a year. Both active and passive auditory stimulation showed potential for enhancing cognitive function in individuals with AD.

**Conclusion:**

The literature suggests that auditory stimulation may positively influence cortical wiring and enhance cognitive abilities. Multimodal interventions that combine auditory stimulation with other sensory or behavioural approaches could yield more substantial effects on global cognition. However, the study design, intervention characteristics and outcome measures varied across studies, underscoring the necessity for standardised reporting. Well-designed studies using standard cognitive assessment protocols are recommended.

**Supplementary Information:**

The online version contains supplementary material available at 10.1186/s13195-024-01544-2.

## Introduction

Alzheimer's disease (AD) is a neurodegenerative disorder that gradually affects an individual's thinking and sociobehavioural skills, significantly reducing their quality of life [1]. Neural degeneration in AD occurs in three stages, with the prodromal stage, i.e., mild cognitive impairment (MCI), primarily impacting the basal regions of the frontal and temporal lobes. Deeper brain areas become increasingly affected in the later stages as the disease progresses [2, 3]. The temporal lobe plays a significant role in auditory signal processing and is implicated in this degenerative process. Previous studies have suggested that auditory processing (AP) deficits precede the clinical symptoms of AD by at least 10 years [4–7].

Behavioural interventions such as cognitive training, physical exercise, lifestyle modifications, meditation, mindfulness, and psychomotor stimulation are commonly used in managing AD, especially in its prodromal stages [8–10]. Processing complex auditory signals often requires greater cognitive skills; hence, auditory stimulation is also suggested as a rehabilitation method for improving cognition [11]. Studies on healthy older adults suggest that auditory training can improve cognitive function, particularly attention and working memory [12, 13]. However, the potential benefits of auditory training have not been much explored in individuals with prodromal AD.

In a conventional auditory training regimen, active participation is typically required of individuals. Recent studies have revealed that passive auditory and visual stimulation at a specific frequency (such as 40 Hz) may induce changes in cortical wiring and enhance cognitive abilities [14]. Monteiro et al. (2021) reviewed studies investigating the motor and cognitive effects of multimodal sensory stimulation in people with cognitive decline or AD [15]. It specifically focused on passive auditory stimulation, and at the time, there were only three human studies on passive auditory stimulation in people with AD [16–18].

Literature suggests that even active auditory stimulation is effective in enhancing cognitive function for individuals with prodromal AD [4]. The addition of recent research and growing interest in this area led us to undertake a comprehensive literature review that included both active and passive auditory training. This review summarised the current state of knowledge on auditory-based interventions, both active and passive, in individuals with AD or its prodromal stages. The objectives were to identify the types of auditory stimulation used, the outcome measures used, and the effects of such stimulation on cognitive function.

## Methodology

To map the different types of auditory stimulation, this scoping review used the five-step framework of Arksey and O’Malley [19]. The five steps include a) formulating the research question, b) searching for literature, c) selecting eligible studies, d) data charting, and e) collating, summarising, and analysing the data. The Preferred Reporting Items for Systematic Reviews and Meta-Analyses extension for Scoping Reviews Checklist (PRISMA-ScR) guidelines [20] were followed to perform the scoping review. The review protocol was developed and registered on the Open Science Framework (OSF), which can be accessed at https://doi.org/10.17605/OSF.IO/89YZ2.

### Step 1: identifying the research question

The review team used the Population, Intervention, and Outcome (PIO) framework to construct the research question (Table 1): What are the existing auditory stimulation techniques used to treat cognition in individuals with Alzheimer's disease and its prodromal stages?

**Table 1:** Eligibility criteria

	**Inclusion criteria**	**Exclusion criteria**
*Population*	Studies having participants diagnosed with Alzheimer’s disease or its prodromal stages, i.e., mild cognitive impairment. Participants of both gender and age range of 55to 90 years	Studies using animal models of Alzheimer’s disease. Studies including individuals with cognitive decline derived from other diseases/conditions (e.g., stroke, ischemia, Parkinson’s disease)
*Intervention*	Auditory-based intervention or stimulation (both active and passive) either alone or combined with other sensory/noninvasive/behavioral intervention techniques.	Studies including other (nonauditory) sensory stimuli as a standalone treatment.
*Outcome*	Outcome measures related to cognitive function/neurophysiological changes using Electroencephalogram (EEG)/improvements in Auditory skills.	-
*Study design*	Study designs include experimental, quasi-experimental, or observational studies in English, encompassing crossover studies, longitudinal studies, randomized and nonrandomized clinical trials, pre- and post-experimental studies, case‒control studies, cohort studies, and case series.	Reviews, protocols, conference papers, proceedings papers, editorials, and surveys

### Step 2: searching for literature

A comprehensive search strategy was developed after discussion with subject experts (HP, KG) and consideration of relevant recent reviews. The possible literature sources were identified by searching the following databases prior to the search date of July 10, 2023: PubMed (NCBI), Web of Science (Clarivate), CINAHL (EBSCO), and Embase (Elsevier). The search was conducted by one of the authors (DSP) using the search terms "Alzheimer’s Disease", "Cognitive Dysfunction", "Cognitive Impairment", "Auditory Rehabilitation", "Auditory Training", "Sound Stimulation", "Gamma Entrainment", "Cognition", "Working Memory", and "Attention", integrating MESH terms where applicable. These keywords were combined using Boolean operators to develop the search strategy (see Appendix 1).

### Step 3: selecting eligible studies

All the identified citations were collected and imported to Covidence systematic review software (accessible at www.covidence.org), with duplicates subsequently removed. Two reviewers conducted a two-stage article review process to mitigate bias or errors. Initially, reviewers (LT, KG) independently screened titles and abstracts, excluding articles that did not meet the criteria. Any disagreements were resolved by a third reviewer (HP). In the second stage, both reviewers examined the full texts, with conflicts resolved by a third reviewer.

### Step 4: charting the data

A predetermined data charting format was used for data extraction by one of the reviewers (LT). Relevant data on country/region, study design, sample characteristics, recruitment site, auditory stimulation techniques, intervention characteristics (duration and frequency), and outcome measures used were extracted. The extracted data were cross-verified by the review team (KG and HP).

### Step 5: collecting, summarizing, and reporting results

The findings are summarized in a narrative way aided by tables where appropriate. The results include details on the study characteristics (study setting and design), participant characteristics (study sample, age range), intervention characteristics (type, equipment used, duration and frequency) and outcome measures.

## Results

**Fig. 1:**
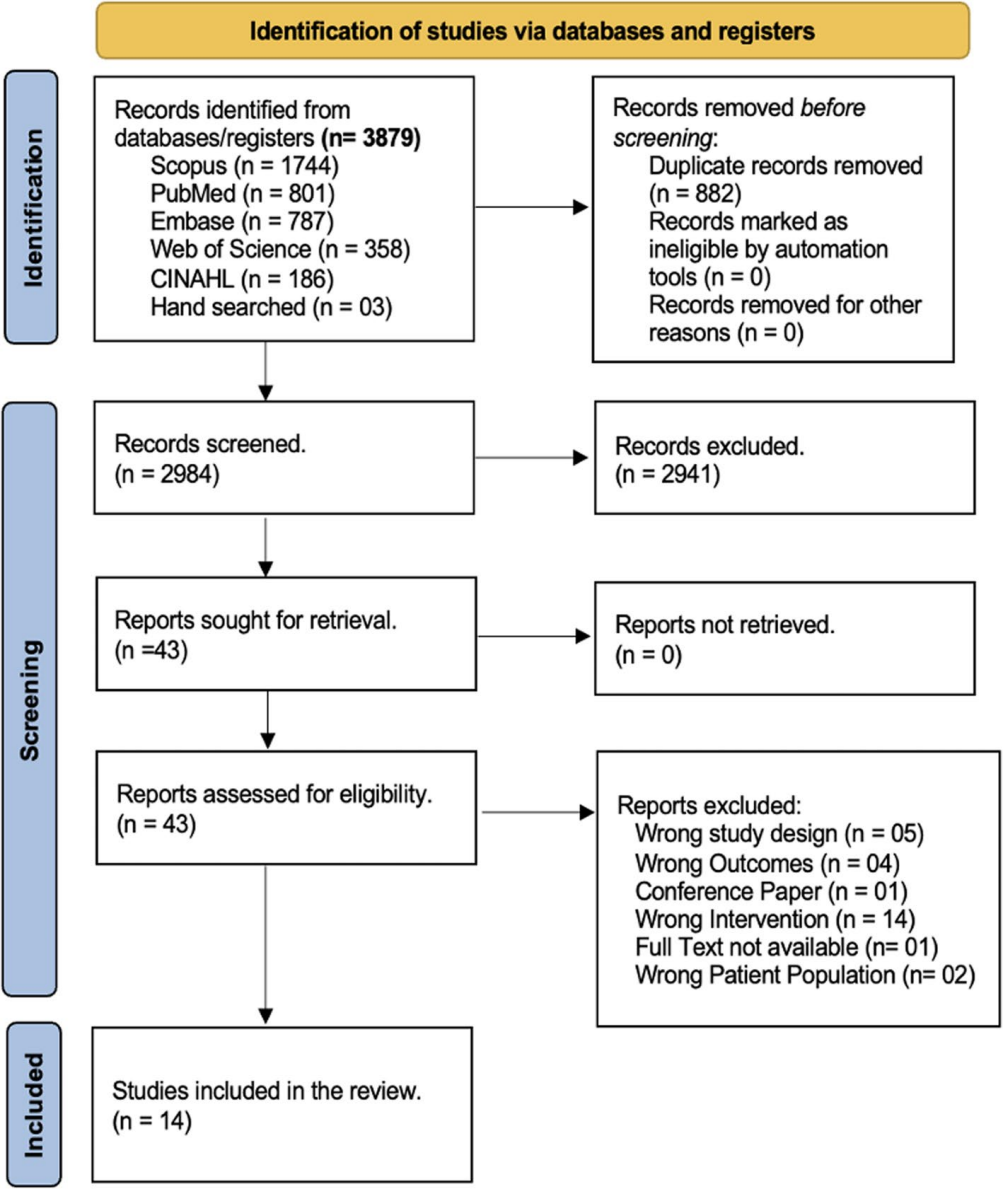
Flowchart of the article selection process

The search yielded a total of 3,879 articles. After removing duplicates (*n* = 882), conducting title and abstract screening (*n* = 2,984), and performing full-text screening (*n* = 43), fourteen articles were ultimately included in the analysis. The PRISMA flow diagram below reports the reasons for excluding articles (see Fig. 1).

### Characteristics of the included studies

#### Study settings

Six of the fourteen studies were conducted in the United States, three in Greece, two each in Canada and China, and one in Brazil.

#### Study designs

The included studies were published between 2009 and 2022. The research designs of these studies included eight randomized controlled trials [16, 18, 21–26], four quasi-experimental studies [17, 27–29], a case series [30] and a case report [31].

#### Participant characteristics

Participants were predominantly recruited from clinic or hospital settings and/or through services for community-dwelling older people. Participants in the study were aged between 59 and 90 years, with diagnoses of either AD or MCI across both sexes and no specific sex ratio requirement. Several different diagnostic criteria have been used to identify cognitive impairment in participants. To recruit individuals with MCI, most studies followed the diagnostic criteria of Peterson et al. published in 1999 [32] [18, 23, 26–29]. Barnes et al. (2009) [21] followed the recommendations of an international consensus committee [33], and Lee et al. (2017) [25] used the Montreal Cognitive Assessment (MoCA) [34]. A few studies recruited individuals who were already diagnosed with AD/MCI and were receiving medication [17, 22, 24, 30, 31]. Clements-Cortes et al. (2016) [16] followed the clinical standards published by the National Institute on Aging and Alzheimer’s Association (NIA-AA) [35].

#### Intervention characteristics

Two categories of auditory stimulation interventions existed in the included studies: active auditory stimulation (7 studies) and passive auditory stimulation (7 studies).

##### Active auditory stimulation

This review identified seven studies that used active auditory stimulation intending to improve cognitive function in individuals with MCI. Active auditory stimulation included the use of computerized software called the Brain Fitness Program (BrainHQ by Posit Science) (as shown in Table 2). Four studies used the BrainHQ for auditory stimulation [21, 23, 25, 26]. This brain fitness program comprises various exercises targeted to enhance the speed and accuracy of AP. The exercises included time order judgment, syllable discrimination, and adaptive recognition of verbal instructions. The other three studies in this category used the BrainHQ along with physical exercise for research conducted in Greece, which was named long-lasting memories (LLM) [27–29]. LLM involves a computerized game-based physical exercise designed particularly for older adults, along with BrainHQ.

**Table 2:** Details of seven studies using active auditory stimulation

**#**	**Author and Year**	**Country**	**Study Design**	**Study Population**	**Intervention Details**	**Frequency and Duration**	**Primary Outcome Measures**
1	Barnes et al. 2009 [21]	United States	RCT	MCI	BrainHQ	100 min/day 5 days/week Till 80% of training is completed	1. RBANS (Repeatable Battery for Assessment of cognitive Status) 2. California Verbal Learning Test II (CVLT-II) 3. Controlled Oral Word Association Test (COWAT) 4. Boston Naming Test (BNT) 5. California Trail Making Test 6. Design Fluency tests from the Delis-Kaplan Executive Function Scale 7. Spatial Span test
2	Rosen et al. 2011 [26]	United States	RCT	MCI	BrainHQ	100 min/day 5 days/week Till 80% of training is completed	1. RBANS (Repeatable Battery for Assessment of cognitive Status) 2. Auditory verbal task during fMRI
3	Chandler et al. 2017 [23]	United States	RCT	aMCI	BrainHQ	10 h in total	1. Dementia rating scale-2 (DRS-2) 2. Mini Mental State Examination (MMSE) 3. Everyday cognition 4. Chronic Disease Self-Efficacy Scales (adapted selected items) 5. Adherence assessment
4	Lee et al. 2017 [25]	China	RCT	Risk of cognitive decline	Training program modeled after Brain fitness (other details not mentioned)	13 weeks	1. Seashore Rhythm Test 2. Digit Vigilance Test 3. Digit span test and Spatial span test
5	Styliadis et al. 2015 [29]	Greece	Pre and Post Experimental design	MCI	LLM- BrainHQ + Physical Exercise	One hour/session (BrainHQ) 3 to 5 days/week 8 weeks	1. MMSE 2. Resting state EEG with eyes closed
6	Bamidis et al. 2015 [27]	Greece	Pre and Post Experimental design	Cognitively healthy to MCI or Dementia	LLM- BrainHQ + Physical Exercise	Ranged from 24 to 110 sessions for 6 weeks	1. California Verbal Learning Test 2. Digit Span Test 3. Trail Making Test
7	Klados et al. 2016 [28]	Greece	Pre and Post Experimental design	MCI	LLM- BrainHQ + Physical Exercise	One hour/session (BrainHQ) 3 to 5 days/week 8 weeks	1. Resting state EEG with eyes closed 2. California Verbal Learning Test 3. Digit Span Test 4. Trail Making Test

##### Passive auditory stimulation

Seven studies utilized passive auditory stimulation. However, only one of them employed solely acoustic stimulation, while the remaining six studies used acoustic stimulation in combination with other sensory stimuli, including light, tactile, and transcranial alternating current stimulation (tACS) (Table 3). Papalambros et al. (2019) [18] employed a phase-locked loop (PLL), a method used in neuroscience to deliver acoustic stimulation in synchrony with brain waves [36]. This involved using EEG to monitor the natural slow-wave oscillations of individuals with MCI during nonrapid eye movement sleep (NREM) stages. Next, brief pink noise pulses were generated in synchrony with specific phases of the recorded brain waves. The pink noise pulses were transmitted through headphones at the most comfortable level (MCL) so as not to disturb the participants' sleep. This acoustic stimulation delivered over one night enhanced slow-wave activity and, in turn, memory recall in individuals with aMCI.

**Table 3:** Details of the seven studies using passive auditory stimulation

**#**	**Author and Year**		**Country**	**Study Design**		**Study Population**		**Intervention Details**	**Frequency and Duration**	**Device Used**	**Primary Outcome Measures**
1	Papalambros et al. 2019 [18]		United States	Randomized Crossover sham controlled study design		aMCI		Phase locked loop acoustic stimulation. (Acoustic tone of 1 Hz phase- locked with endogenous SWA)	1 session (8 h- overnight) Crossover- Sham- 1 week apart	**_**	1. Polysomnography recording along with EEG during sleep 2. Auditory Event- Related Potentials (ERPs) 3. Declarative memory test
2	Calomeni et al. 2017 [17]		Brazil	Pre and post experimental		AD		Brain stimulation by light and sound	10 sessions 15 min/session for 20 days	_	. Alpha and Sensory Motor Rhythm monitoring, 2. Digit span test 3. MMSE
3	He et al. 2021 [24]		United States	Delayed start trial		MCI		Flicker exposure of light and sound	1 h/day 4 or 8 weeks (randomized)	Gamma sense stimulation system by Cognito therapeutics	1. Resting state EEG 2. fMRI 3. Immune factors, AB42, t-tau, and p- tau changes in CSF
4	Chan et al. 2022 [22]		United States		Phase 1- Experimental (single session) Phase 2- Single Blinded RCT	Probable AD	Light and Sound		1 h/day for 6 months	GENUS	1. EEG during stimulation 2. fMRI during FNA-DRT- Face Name Association delayed recall task 3. Neuropsychological battery including MMSE, MoCA, TMT A&B, Craft 21 story recall: immediate and delayed, Digit span- forward and backward, GDS, Fuctional assessment scale, Neuropsychiatric Inventory Questionnaire (NPI-Q), CDR and ADAS- Cog
5	Clements- Cortes et al. 2022 [30]		Canada		Case series report	2 AD and 1 MCI and their partners/caregivers	Multisensory stimulation (40 Hz and tactile stimulation)		30 min/day 5 times/week for 1 year	Sound Oasis VTS 1000	SLUMS- Saint Louis University Mental Status
6	Clements- Cortes et al. 2016 [16]		Canada		Crossover RCT- 2 days washout period	AD	RSS- Rhythmic Sensory Stimulation (Low-frequency sensory stimulation (LFSS) plus vibroacoustic Therapy (VAT))		35–45 min 2 times/week for 6 weeks	40 Hz VAT- NextWave chair Auditory Stimulation software- PhysAc.Net	SLUMS
7	Liu et al. 2022 [31]	China		Case report		AD	tACS combined with sound stimulation tACS: 40 Hz, 1.5 mA. Two electrodes- Dorsolateral prefrontal cortex and contralateral supraorbital area Sound: 40 Hz, 60 dB pure tone through earbuds		15 sessions 20 min/session for 3 weeks	_	1. ADAS- COG 2. MoCA 3. MMSE 4. CDR 5. AVLT- Auditory Verbal Learning Test 6. BAI- Beck Anxiety Inventory

Six articles employed passive auditory stimulation along with other sensory or noninvasive stimulation. Among them, three studies used light stimulation in combination with auditory stimulation. Chan et al. (2022) [22] utilized the Gamma Entrainment Using Sensory Stimulation (GENUS) device from the Picower Institute (https://picower.mit.edu/innovations-inventions/genus), and He et al. (2021) [24] employed the Gamma Sense Stimulation system from Cognito Therapeutics (https://cognitotx.com/). Calomeni et al. (2017) [17] investigated the synergistic effects of light and binaural beats using the Brain Wave Synthesizer named SIRIUS by Mind Place Center, Canada. The study employed a multimodal approach, sequentially combining visual and auditory stimulation with binaural beats and working memory training. However, specific details regarding stimulation parameters and duration were not provided.

Additionally, two studies combined tactile stimulation with auditory stimulation. In the study by Clements-Cortes et al. (2016) [16], participants with AD were randomized into two groups using a crossover design, with a wash-out period between sessions. The sessions comprised either 30 min of 40 Hz rhythmic sensory stimulation (RSS) or visual stimulation. The NextWave chair delivered the RSS via 40 Hz sinusoidal sound waves, providing vibrotactile stimulation across the body. Following a washout period, participants underwent visual stimulation while seated on the NextWave chair. The chair remained inactive, prompting them to engage with visual stimuli such as ocean waves and nature images on a television screen. Another study by Clements-Cortes and Bartel (2022) [30] detailed the experiences of three participants (two with MCI and one with AD) and their caregivers following multisensory gamma stimulation. The intervention involved auditory stimulation with isochronous sound at 40 Hz and tactile stimulation at 40 Hz, delivered through the Sound Oasis VTS 1000.

Finally, Liu et al. (2022) [31] combined tACS at gamma frequency (40 Hz) and sound stimulation simultaneously. The sound stimulation at 40 Hz was delivered through two sponge earbuds placed in the patient’s ears and synchronized with tACS. The tACS was administered using two electrodes positioned at the left dorsolateral prefrontal cortex (F3) and the contralateral supraorbital area (F4).

### Intervention frequency and duration

The duration and frequency of stimulation varied across studies, ranging from a minimum of one session lasting 8 h to 30-min sessions conducted over a year (3 to 5 days per week). The most prevalent approach for active and passive stimulation involved one-hour sessions held 3 to 5 days per week for 8 weeks.

### Outcome measures

Objective measures, such as EEG and/or functional magnetic resonance imaging (fMRI), were utilised in three studies [22, 24], one study employed auditory event-related potentials (ERPs) [18]. Other behavioural outcome measures included MMSE, MoCA, the Dementia Rating Scale (DRS), the Trail Making Test (TMT) A & B, the Digit Span Test (DST), the National Institutes of Health (NIH) Toolbox Cognition Battery, Saint Louis University Mental Status (SLUMS) and several behavioural and neuropsychological tests.

### Effect on cognitive function

Overall, the findings indicate that active auditory training had a positive impact on cognitive function. Several studies have reported improvements in overall cognition [26, 27], as well as in specific cognitive domains such as delayed memory [21], spatial span test [25, 26], CVLT-II [27], TMT [27], and DST [17, 25, 27]. However, Chandler et al. (2017) [23] reported no improvements in any cognitive measures among participants in a brain fitness program.

Passive auditory stimulations, on the other hand, resulted in improvements in cognitive measures such as ADAS-Cog, MMSE, MoCA, AVLT [37], DST [17], face-name association task [22] and SLUMS score [16, 30]. A study by Papalambros et al. (2019) did not show significant improvement in cognitive tests used, such as the verbal paired association test and NIHTB [18].

Studies by Klados et al. (2016) [28] and He et al. (2021) [24] did not directly assess cognition using neuropsychological or behavioural tests; instead, they employed electrophysiological measures such as ERPs, resting-state EEG, and/or fMRI. Results of active intervention using resting-state EEG indicated heightened EEG band activity, particularly in the beta band [28] and the theta band [29]. In addition, fMRI revealed enhanced functional connectivity in the default mode network (DMN) following passive stimulation of light and sound [24].

## Discussion

This scoping review aimed to synthesize the existing evidence on auditory-based interventions for individuals with AD and its prodromal stages. This review identified two primary categories of auditory interventions: active auditory stimulation and passive auditory stimulation. A significant proportion of the included studies adopted a combined modality approach, integrating auditory stimulation with other sensory or behavioural interventions.

### Auditory stimulation

There are various ways to modulate the neurons, one of which is through auditory stimulation [38]. Studies have shown that passive auditory stimulation can significantly change brain function [39]. This is because auditory stimulation can potentially alter neuronal plasticity by increasing the levels of certain neurotransmitters [40]. These improvements could be due to increased phase locking of cortical neurons (even outside the auditory cortex) in response to external stimuli [41]. Furthermore, a study demonstrated the effectiveness of targeted auditory stimulation in modulating slow-wave activity (SWA), a phenomenon crucial for memory consolidation during the nonrapid eye movement (NREM) stage of sleep [18]. Reduced SWA is associated with age-related memory decline [42]. By presenting the SWA frequency through a transducer during this NREM stage of sleep, it is believed that the SWA can be increased, and memory can be improved.

Both passive and active auditory stimulation can result in neuroplastic changes [38]. In the hearing field, traditional auditory training methods focus on active auditory stimulation, requiring active participation from individuals [43]. BrainHQ software encompasses several sets of exercises with different elements to improve cognition, one of which is an auditory module designed to improve speed and accuracy in AP. Auditory processing deficits commonly precede the clinical symptoms of AD [7], and training using the BrainHQ has demonstrated efficacy in enhancing various cognitive skills, including attention, working memory, and language abilities [44]. Generally, active auditory training has resulted in medium to large cognitive enhancement effects in individuals with MCI.

### Multimodal stimulation

Cognitive processes are closely interconnected with various physiological and neural systems. Interventions addressing multiple components and mechanisms through a multimodal approach may yield more substantial effects on global cognition [45]. This review identified studies combining physical exercise with active auditory stimulation, i.e., using BrainHQ software (Auditory component). Exercise promotes synaptic plasticity and neurogenesis by increasing the levels of growth factors such as brain-derived neurotrophic factor (BDNF) and insulin-like growth factor-1 (IGF-1) [46]. Additionally, physical exercise can increase hippocampal size and decrease amyloid deposition, contributing to improved cognitive functions [46, 47]. This review also identified studies that have explored the application of passive auditory stimulation in conjunction with light or other sensory inputs. These studies target sensory entrainment processes, aiming to synchronize neural network rhythms with external stimuli, potentially modulating brain oscillations and altering memory functions [48, 49]. Studies have focused on 40 Hz stimulation, as the results from preclinical studies demonstrated the ability of stimulation at this frequency to reduce the accumulation of β-amyloid plaques, a hallmark feature of AD, in animal models [50, 51]. Recent research aimed to understand the neurobiological mechanisms of sensory entrainment and optimize its therapeutic effectiveness for Alzheimer's disease and related neurodegenerative conditions [52, 53].

### Study design

Studies utilising active auditory stimulation predominantly adopted stronger study designs, with four RCTs [21, 23, 25, 26] and three pre- and post-experimental designs [27–29]. Passive auditory stimulation studies included case reports [31], case series [30], a few RCTs [16, 18, 22, 24] and a pre-post experimental design [17]. It is noticed that research methodologies employed for passive auditory stimulation with two studies being case reports and case series. To strengthen support for the effectiveness of passive auditory stimulation, further research, with a strong study design and adequate sample, is needed.

### Outcome measures

The review revealed a range of outcome measures employed in the identified studies, including objective measures such as EEG and fMRI, along with various behavioural assessments. The lack of consistency in these measures, coupled with variations in intervention duration, poses a challenge in determining the most effective protocols and poses a challenge for future meta-analyses. Standardisation of outcome measures and intervention protocols would facilitate more robust comparisons and meta-analyses, ultimately advancing our understanding of the potential benefits of auditory interventions in individuals with AD. Additionally, considering the baseline/premorbid abilities of participants, such as their variability in AP abilities, could enhance the precision and efficacy of interventions.

### Strengths and limitations

The strengths of this review are that we conducted a comprehensive literature search encompassing different databases over a wide period of time and were able to identify studies utilising a range of auditory stimulation techniques. However, a limitation is the inclusion of only English language studies. Additionally, the authors did not appraise the level of evidence or examine bias in the included studies, as the aim was to identify and synthesise the current evidence to gain an overview of the topic as a basis for future studies.

## Conclusion

Active interventions show potential for improving cognitive function, while passive interventions, especially when combined with other sensory inputs, have the potential to modulate brain oscillations and impact memory functions. To ensure reliable results, it is important to have strong study designs coupled with standardised intervention protocols and outcome measures.

### Supplementary Information

Supplementary Material 1.

## Data Availability

No datasets were generated or analysed during the current study.
