# Supplementary material for: Efficacy of acoustic stimulation techniques on cognitive functions in individuals with Alzheimer’s disease—a scoping review
Source: Alzheimers Res Ther. 2024 Aug 1;16:174. doi: 10.1186/s13195-024-01544-2 (PMC11293131; doi:10.1186/s13195-024-01544-2)
Supplement: Supplementary file 1 — Supplementary Material 1. [file 13195_2024_1544_MOESM1_ESM.docx]

**APPENDIX I**

**Search Strategy**

| **Date of search** |  | **Query** | **No. of articles** |
| --- | --- | --- | --- |
| 10-07-2023 | PubMed | (("Alzheimer Dementia"[Text Word] OR "Alzheimer Dementias"[Text Word] OR "dementia alzheimer"[Text Word] OR "Alzheimer's Disease"[Text Word] OR "dementia alzheimer type"[Text Word] OR "alzheimer type dementia"[Text Word] OR ("alzheimer type dementia"[All Fields] OR "ATD"[Text Word]) OR ("dementia alzheimer type"[All Fields] OR "Alzheimer's Diseases"[Text Word] OR "alzheimer disease*"[MeSH Terms] OR "alzheimer disease*"[Text Word] OR "Alzheimers Diseases"[Text Word] OR "Late Onset Alzheimer Disease"[Text Word] OR "Early Onset Alzheimer Disease"[Text Word] OR ("cognitive dysfunction"[MeSH Terms] OR "Mild cognitive impairment"[Text Word]) OR "Probable AD"[Text Word] OR "Mild Neurocognitive Disorder"[Text Word] OR "disorder mild neurocognitive"[Text Word] OR "disorders mild neurocognitive"[Text Word] OR "Mild Neurocognitive Disorders"[Text Word] OR "Neurodegenerative Disorder"[Text Word])) AND ("Audiologic Rehabilitation"[Text Word] OR "Auditory rehabilitation"[Text Word] OR "Auditory intervention"[Text Word] OR ("acoustic stimulation"[MeSH Terms] OR "auditory stimulation"[Text Word]) OR "Auditory training"[Text Word] OR "Auditory based cognitive training"[Text Word] OR "auditory modality"[Text Word] OR "Sound stimulation"[Text Word] OR "sound stimuli"[Text Word] OR "sensory stimulation"[Text Word] OR "sensory therapy"[Text Word] OR "sound"[Text Word] OR "sound"[MeSH Terms] OR ("brain waves"[MeSH Terms] OR "brain wave"[Text Word]) OR "Gamma Rhythm"[MeSH Terms] OR "Gamma Rhythm"[Text Word] OR "gamma wave*"[Text Word] OR "sensory evoked gamma oscillation"[Text Word] OR "gamma entrainment"[Text Word] OR "entrainment"[Text Word] OR "GENUS"[Text Word] OR "steady state response"[Text Word] OR "40 Hz"[Text Word] OR "isochronic"[Text Word] OR "isochronous"[Text Word] OR "binaural beats"[Text Word]) AND ("cognition*"[MeSH Terms] OR "cognition*"[Text Word] OR "Cognitive Function"[Text Word] OR "Cognitive Functions"[Text Word] OR "electroencephalography"[MeSH Terms] OR "Electroencephalogram"[Text Word] OR "EEG"[Text Word] OR "Electroencephalograms"[Text Word] OR "electroencephalography"[MeSH Terms] OR ("memory, short term"[MeSH Terms] OR "working memory"[Text Word]) OR "attention"[MeSH Terms] OR "attention"[Text Word] OR "Focus of Attention"[Text Word] OR "Social Attention"[Text Word] OR "Auditory Perception"[Text Word] OR "Auditory Perception"[MeSH Terms] OR "Auditory Processing"[Text Word])) AND ((humans[Filter]) AND (english[Filter])) | 793 |
| 10-07-2023 | Web of Science | **#3 AND #2 AND #1 [English language limits applied]**  **#1**  TS=(cognition* OR "cognitive function*" OR "function* cognitive" OR electroencephalography OR Electroencephalogram* OR EEG OR "memory, short term" OR "working memory" OR Attention OR "focus of attention" OR "social attention" OR "auditory perception" OR "auditory perceptual disorders" OR "auditory processing")  **#2**  TS=((((((((((((((((((((((((((((((((((((((("Audiologic Rehabilitation") OR ("Audiologic Rehabilitations")) OR ("Rehabilitation, Audiologic")) OR ("Auditory rehabilitation")) OR ("Auditory intervention")) OR ("acoustic stimulation" OR "auditory stimulation")) OR ("Auditory training")) OR ("Auditory based cognitive training")) OR ("auditory modality")) OR ("game-based auditory training")) OR ("game based auditory training")) OR ("Sound stimulation")) OR ("sound stimuli")) OR ("sensory stimulation")) OR ("sensory therapy")) OR (sound)) OR ("brain waves" OR "brain wave")) OR ("Gamma Rhythm")) OR ("Gamma Rhythm")) OR ("gamma wave*")) OR ("sensory evoked gamma oscillation")) OR ("gamma entrainment")) OR (entrainment)) OR (GENUS)) OR ("steady state response")) OR (“40 Hz”)) OR (isochronic)) OR (isochronous)) OR ("monoaural beats")) OR ("binaural beats")))))))))))  **#3**  TS=(((((((((((((((((((((((((((((((((((((((((("Alzheimer Dementia")) OR ("Alzheimer Dementias")) OR ("Dementia, Alzheimer")) OR ("Alzheimer's Disease")) OR ("Dementia, Alzheimer Type")) OR ("Alzheimer Type Dementia")) OR ("Alzheimer-Type Dementia" (ATD))) OR ("Alzheimer's Diseases")) OR ("Alzheimers Diseases")) OR ("Late Onset Alzheimer Disease")) OR ("Focal Onset Alzheimer's Disease")) OR ("Early Onset Alzheimer Disease")) OR ("cognitive dysfunction" OR "Mild cognitive impairment")) OR ("Probable AD")) OR ("Prodromal AD")) OR ("Mild Neurocognitive Disorder")) OR ("Disorder, Mild Neurocognitive")) OR ("Disorders, Mild Neurocognitive")) OR ("Mild Neurocognitive Disorders")) OR ("Neurodegenerative Disorder"))))))))))))))))))))))) | **358** |
| 10-07-2023 | CINAHL | **#1 AND #2 AND #3 [Humans and English language limits applied]**  **#1**  ((((((((((((((((((((((((((((((((((((((((("Alzheimer Dementia") OR ((MH "Alzheimer Dementia+"))) OR ((MH "Alzheimer Dementias+"))) OR ("Alzheimer Dementias")) OR ("Dementia, Alzheimer")) OR ((MH "Dementia, Alzheimer+"))) OR ((MH "Alzheimer's Disease+"))) OR ("Alzheimer's Disease")) OR ("Dementia, Alzheimer Type")) OR ((MH "Dementia, Alzheimer Type+"))) OR ((MH "Alzheimer Type Dementia+"))) OR ("Alzheimer Type Dementia")) OR ("Alzheimer-Type Dementia" (ATD))) OR ("Alzheimer's Diseases")) OR ((MH "Alzheimer's Diseases+"))) OR ((MH "Alzheimer Disease*+"))) OR ("Alzheimer Disease*")) OR ("Alzheimers Diseases")) OR ((MH "Alzheimers Diseases+"))) OR ((MH "Late Onset Alzheimer Disease+"))) OR ("Late Onset Alzheimer Disease")) OR ("Focal Onset Alzheimer's Disease")) OR ((MH "Focal Onset Alzheimer's Disease+"))) OR ((MH "Early Onset Alzheimer Disease+"))) OR ("Early Onset Alzheimer Disease"))) OR ((MH "cognitive dysfunction+") OR "Mild cognitive impairment")) OR ("Probable AD")) OR ("Prodromal AD")) OR ("Mild Neurocognitive Disorder")) OR ((MH "Mild Neurocognitive Disorder+"))) OR ((MH "Disorder, Mild Neurocognitive+"))) OR ("Disorder, Mild Neurocognitive")) OR ("Disorders, Mild Neurocognitive")) OR ((MH "Disorders, Mild Neurocognitive+"))) OR ((MH "Mild Neurocognitive Disorders+"))) OR ("Mild Neurocognitive Disorders")) OR ("Neurodegenerative Disorder")) OR ((MH "Neurodegenerative Disorder+")))))  **#2**  ((((((((((((((((((((((((((((((((((((((MH "Audiologic Rehabilitation+")) OR ("Audiologic Rehabilitation")) OR ("Audiologic Rehabilitations")) OR ((MH "Audiologic Rehabilitations+"))) OR ((MH "Rehabilitation, Audiologic+"))) OR ("Rehabilitation, Audiologic")) OR ("Auditory rehabilitation")) OR ((MH "Auditory rehabilitation+"))) OR ((MH "Auditory intervention+"))) OR ("Auditory intervention")) OR ((MH "acoustic stimulation+") OR "auditory stimulation")) OR ("Auditory training")) OR ("Auditory based cognitive training")) OR ("auditory modality")) OR ("game-based auditory training")) OR ("game based auditory training")) OR ("Sound stimulation")) OR ("sound stimuli")) OR ("sensory stimulation")) OR ("sensory therapy")) OR (sound)) OR ((MH sound+))) OR ((MH "brain waves+") OR "brain wave")) OR ((MH "Gamma Rhythm+"))) OR ("Gamma Rhythm")) OR ("gamma wave*")) OR ("sensory evoked gamma oscillation")) OR ("gamma entrainment")) OR (entrainment)) OR (GENUS)) OR ("steady state response")) OR ("40 Hz")) OR (isochronic)) OR ((MH isochronic+))) OR ((MH isochronous+))) OR (isochronous)) OR ("monoaural beats")) OR ("binaural beats")  **#3**  (MH cognition*+) OR "cognitive function*" OR "function* cognitive" OR (MH electroencephalography+) OR Electroencephalogram* OR EEG OR (MH "memory, short term+") OR "working memory" OR (MH Attention+) OR "focus of attention" OR "social attention" OR (MH "auditory perception+") OR (MH "auditory perceptual disorders+") OR "auditory processing" | 186 |
| 10-07-2023 | Embase | **#7 AND #9 AND #13 AND [humans]/lim AND [english]/lim**  **#7**  cognition:ab OR 'cognitive function*' OR 'cognitive function*':ab OR 'electroencephalogram'/exp OR 'electroencephalogram' OR electroencephalogram:ab OR 'eeg'/exp OR 'eeg' OR eeg:ab OR electroencephalograms:ab OR 'electroencephalography'/exp OR 'electroencephalography' OR electroencephalography:ab OR 'working memory'/exp OR 'working memory' OR 'working memory':ab OR 'attention'/exp OR 'attention' OR attention:ab OR 'auditory perception'/exp OR 'auditory perception' OR 'auditory perception':ab OR 'auditory perceptual disorders'/exp OR 'auditory perceptual disorders' OR 'auditory perceptual disorders':ab OR 'auditory processing'/exp OR 'auditory processing' OR 'auditory processing':ab  **#9**  'alzheimer dementia*':ti OR 'alzheimers disease*':ti OR 'alzheimer type dementia':ti OR 'alzheimer-type dementia':ti OR 'late onset alzheimer disease':ti OR 'focal onset alzheimers disease':ti OR 'early onset alzheimer disease':ti OR 'cognitive dysfunction':ti OR 'mild cognitive impairment':ti OR 'probable ad':ti OR 'prodromal ad':ti OR 'mild neurocognitive disorder':ti OR 'mild neurocognitive disorders':ti OR 'neurodegenerative disorder':ti  **#13**  'audiologic rehabilitation':ti OR 'audiologic rehabilitations':ti OR 'auditory rehabilitation'/exp OR 'auditory rehabilitation' OR 'auditory rehabilitation':ti OR 'auditory intervention':ti OR 'acoustic stimulation'/exp OR 'acoustic stimulation' OR 'acoustic stimulation':ti OR 'auditory stimulation'/exp OR 'auditory stimulation' OR 'auditory stimulation':ti OR 'auditory training'/exp OR 'auditory training' OR 'auditory training':ti OR 'auditory based cognitive training':ti OR 'auditory modality':ti OR 'game-based auditory training':ti OR 'game based auditory training':ti OR 'sound stimulation'/exp OR 'sound stimulation' OR 'sound stimulation':ti OR 'sound stimuli':ti OR 'sensory stimulation'/exp OR 'sensory stimulation' OR 'sensory stimulation':ti OR 'sensory therapy':ti OR 'sound'/exp OR 'sound' OR sound:ti OR 'brain waves'/exp OR 'brain waves' OR 'brain waves':ti OR 'brain wave'/exp OR 'brain wave' OR 'brain wave':ti OR 'gamma rhythm'/exp OR 'gamma rhythm' OR 'gamma rhythm':ti OR 'gamma wave*':ti OR 'sensory evoked gamma oscillation':ti OR 'gamma entrainment':ti OR 'entrainment'/exp OR 'entrainment' OR entrainment:ti OR 'genus'/exp OR 'genus' OR genus:ti OR 'steady state response':ti OR '40 hz':ti OR isochronic:ti OR isochronous:ti OR 'monoaural beats':ti OR 'binaural beats':ti | 786 |
| 10-07-2023 | SCOPUS | (INDEXTERMS ( cognition ) OR TITLE-ABS-KEY ( cognition ) OR TITLE-ABS-KEY ( "cognitive function*" ) OR INDEXTERMS ( electroencephalography ) OR TITLE-ABS-KEY ( electroencephalogram ) OR TITLE-ABS-KEY ( eeg ) OR TITLE-ABS-KEY ( electroencephalograms ) OR TITLE-ABS-KEY ( "working memory" ) OR INDEXTERMS ( attention ) OR TITLE-ABS-KEY ( attention ) OR TITLE-ABS-KEY ( "auditory perception" ) OR INDEXTERMS ( "auditory perception" ) OR INDEXTERMS ( "auditory perceptual disorders" ) OR TITLE-ABS-KEY ( "auditory processing" ) ) AND ( ( ( ( ( ( ( ( ( ( ( ( ( ( ( ( ( ( ( ( ( ( ( ( ( ( ( ( ( ( ( ( ( ( ( ( ( ( INDEXTERMS ( "Audiologic Rehabilitation*" ) ) OR ( TITLE-ABS-KEY ( "Audiologic Rehabilitation*" ) ) ) OR ( TITLE-ABS-KEY ( "Auditory rehabilitation" ) ) ) OR ( INDEXTERMS ( "Auditory rehabilitation" ) ) ) OR ( INDEXTERMS ( "Auditory intervention" ) ) ) OR ( TITLE-ABS-KEY ( "Auditory intervention" ) ) ) OR ( INDEXTERMS ( "acoustic stimulation" ) OR TITLE-ABS-KEY ( "auditory stimulation" ) ) ) OR ( TITLE-ABS-KEY ( "Auditory training" ) ) ) OR ( TITLE-ABS-KEY ( "Auditory based cognitive training" ) ) ) OR ( TITLE-ABS-KEY ( "auditory modality" ) ) ) OR ( TITLE-ABS-KEY ( "game-based auditory training" ) ) ) OR ( TITLE-ABS-KEY ( "game based auditory training" ) ) ) OR ( TITLE-ABS-KEY ( "Sound stimulation" ) ) ) OR ( TITLE-ABS-KEY ( "sound stimuli" ) ) ) OR ( TITLE-ABS-KEY ( "sensory stimulation" ) ) ) OR ( TITLE-ABS-KEY ( "sensory therapy" ) ) ) OR ( TITLE-ABS-KEY ( sound ) ) ) OR ( INDEXTERMS ( sound ) ) ) OR ( INDEXTERMS ( "brain waves" ) OR TITLE-ABS-KEY ( "brain wave" ) ) ) OR ( INDEXTERMS ( "Gamma Rhythm" ) ) ) OR ( TITLE-ABS-KEY ( "Gamma Rhythm" ) ) ) OR ( TITLE-ABS-KEY ( "gamma wave*" ) ) ) OR ( TITLE-ABS-KEY ( "sensory evoked gamma oscillation" ) ) ) OR ( TITLE-ABS-KEY ( "gamma entrainment" ) ) ) OR ( TITLE-ABS-KEY ( entrainment ) ) ) OR ( TITLE-ABS-KEY ( genus ) ) ) OR ( TITLE-ABS-KEY ( "steady state response" ) ) ) OR ( TITLE-ABS-KEY ( hz ) ) ) OR ( TITLE-ABS-KEY ( isochronic ) ) ) OR ( INDEXTERMS ( isochronic ) ) ) OR ( INDEXTERMS ( isochronous ) ) ) OR ( TITLE-ABS-KEY ( isochronous ) ) ) OR ( TITLE-ABS-KEY ( "monoaural beats" ) ) ) OR ( TITLE-ABS-KEY ( "binaural beats" ) ) ) ) ) ) ) AND ( ( ( ( ( ( ( ( ( ( ( ( ( ( ( ( ( ( ( ( ( ( ( ( ( ( ( ( ( ( ( ( ( ( ( ( ( ( ( ( TITLE-ABS-KEY ( "Alzheimer Dementia" ) ) OR ( INDEXTERMS ( "Alzheimer Dementia" ) ) ) OR ( INDEXTERMS ( "Alzheimer Dementias" ) ) ) OR ( TITLE-ABS-KEY ( "Alzheimer Dementias" ) ) ) OR ( INDEXTERMS ( "Alzheimer’s Disease" ) ) ) OR ( TITLE-ABS-KEY ( "Alzheimer’s Disease" ) ) ) OR ( TITLE-ABS-KEY ( "Dementia, Alzheimer Type" ) ) ) OR ( INDEXTERMS ( "Dementia, Alzheimer Type" ) ) ) OR ( INDEXTERMS ( "Alzheimer Type Dementia" ) ) ) OR ( TITLE-ABS-KEY ( "Alzheimer Type Dementia" ) ) ) OR ( "Alzheimer-Type Dementia" ( TITLE-ABS-KEY ( atd ) ) ) ) OR ( TITLE-ABS-KEY ( "Alzheimers Disease*" ) ) ) OR ( INDEXTERMS ( "Alzheimers Disease*" ) ) ) OR ( INDEXTERMS ( "Alzheimer Disease*" ) ) ) OR ( TITLE-ABS-KEY ( "Alzheimer Disease*" ) ) ) OR ( INDEXTERMS ( "Late Onset Alzheimer Disease" ) ) ) OR ( TITLE-ABS-KEY ( "Late Onset Alzheimer Disease" ) ) ) OR ( TITLE-ABS-KEY ( "Focal Onset Alzheimer Disease" ) ) ) OR ( INDEXTERMS ( "Focal Onset Alzheimer Disease" ) ) ) OR ( INDEXTERMS ( "Early Onset Alzheimer Disease" ) ) ) OR ( TITLE-ABS-KEY ( "Early Onset Alzheimer Disease" ) ) ) ) OR ( INDEXTERMS ( "cognitive dysfunction" ) OR TITLE-ABS-KEY ( "Mild cognitive impairment" ) ) ) OR ( TITLE-ABS-KEY ( "Probable AD" ) ) ) OR ( TITLE-ABS-KEY ( "Prodromal AD" ) ) ) OR ( TITLE-ABS-KEY ( "Mild Neurocognitive Disorder*" ) ) ) OR ( INDEXTERMS ( "Mild Neurocognitive Disorder*" ) ) ) OR ( TITLE-ABS-KEY ( "Neurodegenerative Disorder" ) ) ) OR ( INDEXTERMS ( "Neurodegenerative Disorder" ) ) ) ) ) ) ) ) ) ) ) ) ) ) AND ( LIMIT-TO ( LANGUAGE , "English" ) ) | 1737 |
